# Supplementary figures and images for: The Effects and Regulatory Mechanism of Casein-Derived Peptide VLPVPQK in Alleviating Insulin Resistance of HepG2 Cells
Source: Foods. 2023 Jul 7;12(13):2627. doi: 10.3390/foods12132627 (PMC10340617; doi:10.3390/foods12132627)

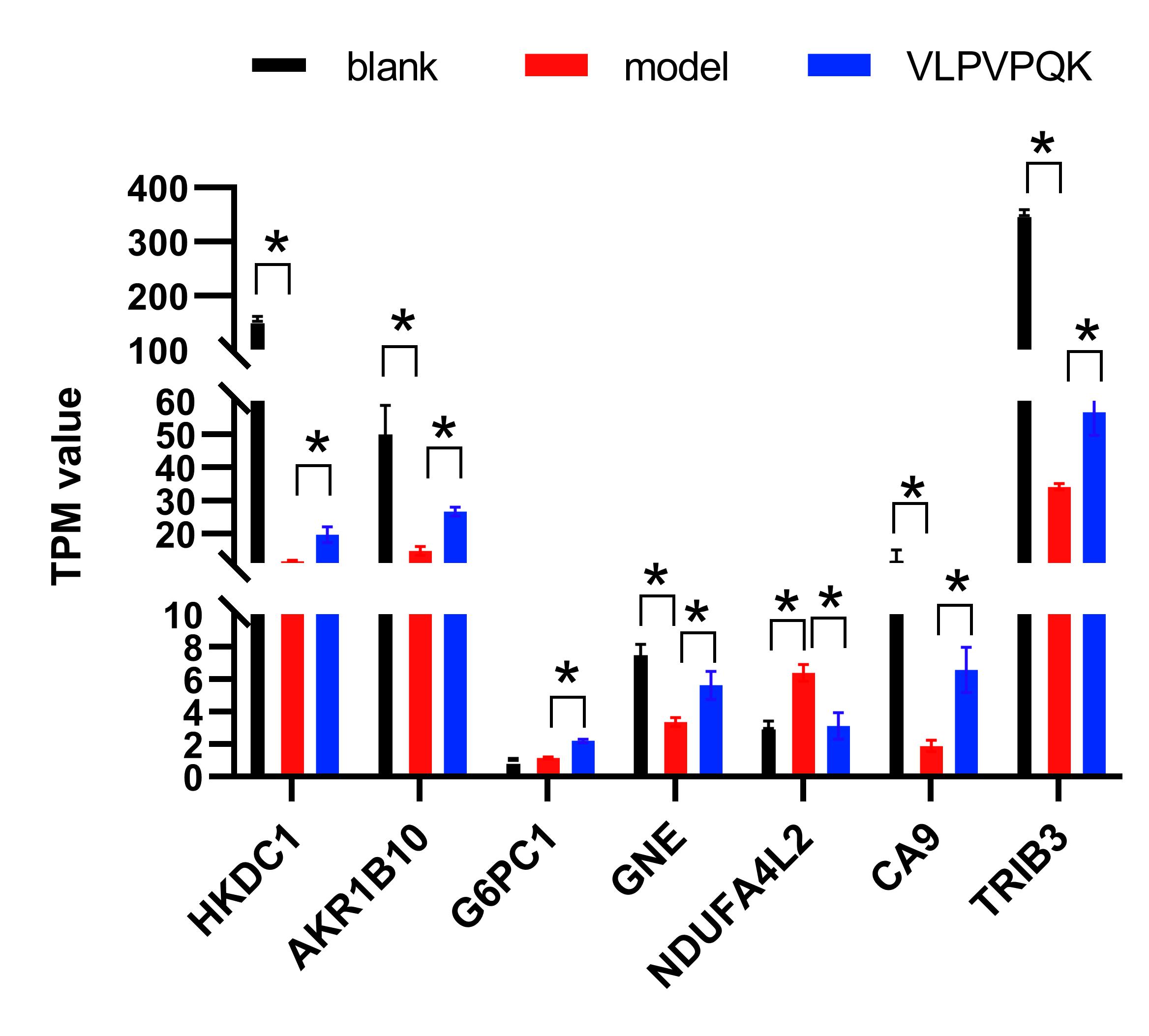

Supplement: Supplementary file 1 [file foods-12-02627-s001.zip › figure.S1 TPM values of 7 genes in model, blank and VLVPVPQK groups.jpg]

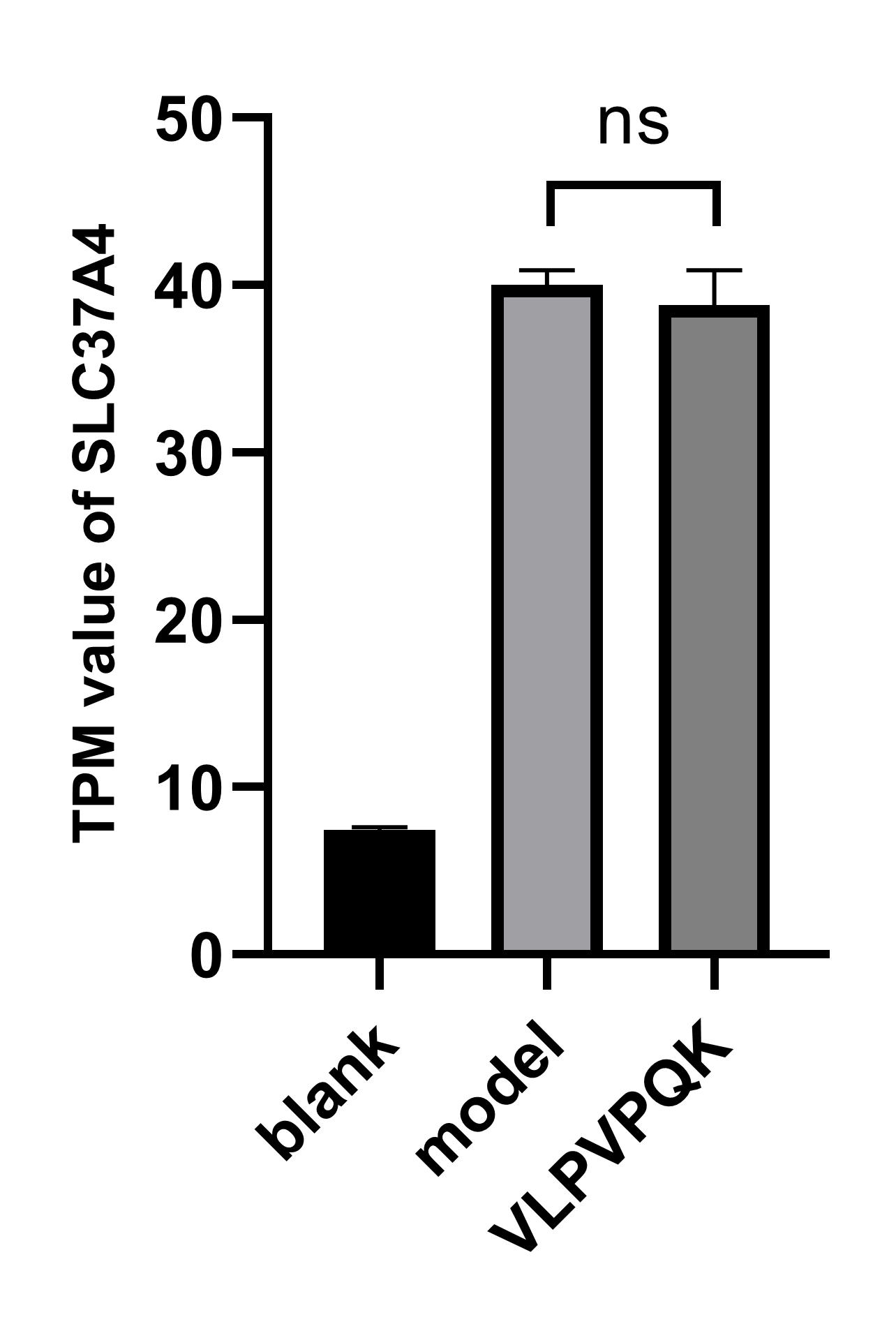

Supplement: Supplementary file 1 [file foods-12-02627-s001.zip › figure.S2 TPM values of SLC37A4 in the model, blank and VLVPVPQK groups.jpg]
